# Dealing With the Aftermath of Mass Disasters: A Field Study on the Application of EMDR Integrative Group Treatment Protocol With Child Survivors of the 2016 Italy Earthquakes

**DOI:** 10.3389/fpsyg.2018.00862

**Published:** 2018-06-04

**Authors:** Cristina Trentini, Marco Lauriola, Alessandro Giuliani, Giada Maslovaric, Renata Tambelli, Isabel Fernandez, Marco Pagani

**Affiliations:** ^1^Department of Dynamic and Clinical Psychology, “Sapienza” University of Rome, Rome, Italy; ^2^Department of Social and Developmental Psychology, “Sapienza” University of Rome, Rome, Italy; ^3^Environment and Health Department, Istituto Superiore di Sanità, Rome, Italy; ^4^EMDR Italy Association, Varedo, Italy; ^5^EMDR Europe Association, Varedo, Italy; ^6^Institute of Cognitive Sciences and Technologies, CNR, Rome, Italy

**Keywords:** EMDR-IGTP, earthquake, mass disaster, children, emotional problems, post-traumatic reactions

## Abstract

This study explored the effects of the EMDR Integrative Group Treatment Protocol (EMDR-IGTP) on child survivors of the earthquakes that struck Umbria, a region of central Italy, on August 24th and on October 26th 2016. Three hundred and thirty-two children from the town of Norcia and nearby severely disrupted villages received 3 cycles of EMDR-IGTP. The Emotion Thermometers (ET-5) and the Children's Revised Impact of Event Scale (CRIES-13) were administered before (T0) and about 1 week after the conclusion of the third cycle (T3) of EMDR-IGTP. At T3, older children showed a reduction of distress and anger, whereas younger children reported an increase on these domains; moreover, older children reported a greater reduction of anxiety than younger ones. A greater reduction of distress, anxiety, and need for help was evidenced in females, whereas a greater improvement in depressive symptoms was evidenced in males. The effects of the EMDR-IGTP treatment on post-traumatic symptoms were particularly evident in older children, compared to younger ones, and marginally greater in females than in males; moreover, a greater improvement was found in children who had received a timelier intervention, than in those who received delayed treatment. These results provide further evidence for the utility of EMDR-IGTP in dealing with the extensive need for mental health services in mass disaster contexts. Also, these data highlight the importance of providing EMDR-IGTP in the immediate aftermath of a natural disaster, to contribute significantly in restoring adaptive psychological functioning in children, especially in older ones.

## Introduction

Scientific literature has provided large evidence for the detrimental psychopathological *sequelae* of natural disasters among children and adolescent survivors. Even though some individuals may show resilience after facing such traumatic experiences and manifest temporary sub-clinical stress responses (Bonanno, 2004), a wide range of psychopathological outcomes has been documented in the exposed population. The prevalence of psychopathological symptoms among child survivors after natural disasters vary largely across studies, according to differences in the implemented methodologies, disaster type and magnitude, as well as in the diagnostic criteria (for a systematic review, see Wang et al., 2013). Nevertheless, severe psychopathological outcomes, such as anxiety, depression, and post-traumatic stress disorder (PTSD) are commonly observed in individuals who are exposed to natural disaster (Liu et al., 2011; Zhang et al., 2011), along with other forms of emotional distress (Toyabe et al., 2006; Oyama et al., 2012), difficulties in regulating anger (Durkin, 1993; Kar and Bastia, 2006; Becker-Blease et al., 2010), and poorer quality of life (Tsai et al., 2007; Jia et al., 2010).

The prompt availability of psychological interventions in the aftermath of a natural disaster has become essential to prevent the onset, as well as the worsening of psychopathological symptoms in exposed individuals (National Institute of Mental Health, 2002; Te Brake et al., 2009), especially in children, who are more vulnerable to the dramatic effects of critical events, compared to adults (Norris et al., 2002b). Indeed, children's psychopathological responses may be enduring (Ularntinon et al., 2008; Piyasil et al., 2011) and persist until adulthood (Honig et al., 1993; Green et al., 1994), with a significant impairment of their individual functioning throughout their lifespan.

The use of relatively brief trauma-focused treatments has relevant implications in the field of mass disaster contexts, where crisis interventions meet the urgent need “to first stabilize and then reduce symptoms of distress or dysfunction, so as to achieve a state of adaptive functioning, or to facilitate access to a continuum of care when necessary” (Everly and Mitchell, 2008, p. 8). The practice guidelines of the World Health Organization (2013) recommend trauma-focused cognitive behavioral therapy (CBT) and Eye Movement Desensitization and Reprocessing (EMDR; Shapiro, 1989) for children, adolescents, and adults manifesting PTSD symptomatology. However, although both treatments have been proven effective in mitigating the effects of PTSD, in a randomized controlled trial study, EMDR resulted in a faster recovery compared with a more gradual improvement provided by CBT (Nijdam et al., 2012). This is due to the fact that, unlike CBT, EMDR does not require extended exposure, does not ask the traumatized individuals to provide detailed descriptions of the event, and does not include direct challenging of beliefs or homework (World Health Organization, 2013). Therefore, these factors make EMDR therapy particularly suitable to rapidly deal with the psychological *sequelae* of a natural disaster.

EMDR has been recommended as a first-line trauma treatment in the international practice guidelines of several organizations, including the American Psychiatric Association (2004) and the Department of Defense Department of Veterans Affairs (2017). The clinical effectiveness of EMDR for treatment of trauma in adults has been broadly documented in about 30 randomized controlled studies, as reported by the EMDR International Association (EMDRIA, retrieved from http://emdria.site-ym.com/?page=Randomized) and an incremental effect of EMDR has been observed in children and adolescents when EMDR was used along with CBT (Rodenburg et al., 2009). Furthermore, in the field of mass disaster contexts, several studies have examined the role of EMDR in alleviating trauma-related symptoms following natural disasters (Grainger et al., 1997; Chemtob et al., 2002; de Roos et al., 2011; Tang et al., 2015). In this domain, research has documented that, although EMDR and CBT are equally able to induce a long-term amelioration of children's disaster-related post-traumatic symptoms, treatment gains of EMDR are reached in fewer sessions (de Roos et al., 2011).

The theoretical model of the Adaptive Information Processing, which guides the EMDR procedures (AIP; Shapiro, 2001), posits that the intense disturbing affect that accompanies trauma causes the information processing system to fail in adequately processing and storing the information (e.g., images, thoughts, emotions, and sensations associated to the traumatic event) into functional memory networks. The eight-phased EMDR protocol aims at accessing these dysfunctionally stored information and facilitates the integration of traumatic memories, leading to their adaptive resolution (Shapiro, 2012). Throughout the 8 EMDR phases, the person is asked to focus on his/her traumatic memories (*target*), while simultaneously being exposed to alternating bilateral stimulation (i.e., eye movements, tactile taps, or auditory tones).

In the last years, several theoretical models have been proposed to account for the mechanisms of action involved in EMDR: among them, the *working memory theories* and the *orienting response theory* appear particularly interesting. According to the *working memory theories* of EMDR, eye movements and visual imagery both draw upon the same limited capacity working memory resources (Baddeley, 2000). The competition created by the dual task performance impairs imagery, causing it to become less vivid and less emotionally intense (Gunter and Bodner, 2008; Maxfield et al., 2008; van den Hout and Engelhard, 2012): as a result, this can facilitate the accessing and processing of the traumatic memory from a more observational or detached perspective, since the person experiences it as less distressing (Maxfield et al., 2008). According to the *orienting response theory* of EMDR, eye movements activate an “investigatory reflex,” which at first induces a state of heightened alertness, and subsequently a reflexive pause, leading to de-arousal in the absence of threat, allowing cognitive processes to become more flexible and efficient (Armstrong and Vaughan, 1996; Kuiken et al., 2001; Lee and Cuijpers, 2013). In addition to these theoretical perspectives, more recent outcomes from electroencephalographic (Pagani et al., 2011, 2012; Trentini et al., 2015) and neurobiological findings (Pagani et al., 2017) have proposed that bilateral stimulation might reproduce the neurophysiological conditions favorable for memory consolidation, weakening the perception of the traumatic memory, reducing its vividness, and inducing a sense of relaxation and safety.

Several modified EMDR protocols have been developed to tailor EMDR procedures to the processing of traumatic experiences in individuals who reported acute traumatic stress. Among these adjusted protocols, the EMDR Integrative Group Treatment Protocol (EMDR-IGTP) results particularly useful to quickly restore psychological functioning in large groups of survivors of natural disaster. EMDR-IGTP was developed by the members of the Asociación Mexicana para Ayuda Mental en Crisis (AMAMECRISIS) to respond rapidly to the need for mental health interventions, after the 1997 hurricane Pauline that struck the Western coast of Mexico. The EMDR-IGTP takes the wisdom of the Standard EMDR Protocol and applies it in an adapted form, together with a group therapy model, an art therapy format, and the use of the Butterfly Hug (BH), which is a form of self-administered bilateral stimulation (Boel, 1999; Artigas et al., 2000; Artigas and Jarero, 2009; Jarero et al., 2012). This protocol was originally designed within a play therapy format with children and was modified later for its application with adults. The EMDR-IGTP has been largely used in its original format or with some adjustments according to different cultural circumstances, to fulfill the need of post-disaster psychological interventions of survivors of natural or man-made disasters, in numerous places around the world (Jarero et al., 2012; for an extensive review, see http://emdrresearchfoundation.org/toolkit/igtp-children.pdf).

On August 24th and on October 30th 2016, two earthquakes (of 6.0 and 6.5 Richter scale magnitude; retrieved from http://cnt.rm.ingv.it/events?starttime=2016-08-24%2B00%253A 00%253A00&endtime=2016-10-31%2B23%253A59%253A59& last_nd=-1&minmag=4&maxmag=10&mindepth=-10&maxdep th=1000&minlat=35&maxlat=49&minlon=5&maxlon=20&min version=100&limit=30&orderby=ot-desc&tdmt_flag=-1&lat=0 &lon=0&maxradiuskm=-1&wheretype=area&box_search=Italia &page=1) struck Umbria, a central region of Italy, causing heavy disruption in the town of Norcia, as well as in many surrounding villages. The day after the first earthquake, the Psychologists Order of Umbria, supported by the Civil Protection of Umbria, established a psychological support network for the population, in collaboration with emergency psychologists and the Italian EMDR National Association. A network of EMDR therapists working *pro bono* immediately delivered an EMDR early intervention, as well as ongoing treatment to survivors. An EMDR-IGTP treatment plan was immediately implemented within an extensive on-site emergency psychology program, 1 day following the first earthquake, with an outreach program based on the principles of emergency psychology: (a) reaching out to the affected community and exposure groups; (b) carrying out an initial psychological triage, to assess the severity of psychological problems and emotional disturbances in the population; (c) providing information in written and verbal form about the typical posttraumatic stress reactions; (d) providing written information to the affected community about the availability of on-site psychological support and EMDR therapists; (e) developing an outreach program and linking with the local municipalities, schools and institutions, police forces, as well as health and social services, in order to provide consistent information among the population. Furthermore, professionals were debriefed about the provision of emergency post-disaster services during the acute phase to the affected population (firefighters, policemen, Carabinieri, members of the Red Cross and forest rangers). Special attention was given to schools, delivering timely psychological support to parents, teachers and students, and planning EMDR-ITGP interventions in accordance with the Ministry of Education, Universities and Research (MIUR).

This intervention was a clear example of a successful collaboration among the Italian EMDR National Association, all institutions and local services that contributed in dealing with the emergency in the aftermath. This preliminary study investigated the effects of EMDR-ITGP on emotional problems and post-traumatic symptoms in children who had been exposed to both earthquakes that struck central Italy on August 24th and on October 26th 2016.

## Materials and methods

### Participants

Initially, a total of 701 children were recruited at the schools of Norcia and from the nearby severely damaged villages. The schools provided an opportunity to rapidly recruit children, since many people had been displaced from their homes and were living in container homes and makeshift camps. According to the guidelines of emergency psychology, which strongly recommend to provide all individuals (both, those presenting PTSD symptomatology, as well as those presenting subclinical conditions) with prompt intervention, all recruited children were treated with EMDR-IGTP.

Children received EMDR-IGTP once a week for 3 weeks (that is, 3 EMDR-IGTP cycles) and were tested before (T0) and about 1 week after the conclusion of the third treatment cycle (T3). As agreed with the school administrators, and in order to restore normal school routine as quickly as possible, children received only 3 cycles of EMDR-IGTP.

Children who did not complete all EMDR-IGTP cycles (*N* = 369) were excluded from the statistical analyses; thus, the final sample included 332 children, aged between 5 and 13 (Mean = 9.15, Standard Deviation = 2.31).

This study has been carried out in accordance with the recommendations of the Ethics Committee of the Institute of Cognitive Sciences and Technologies of the Italian National Research Council (ISTC-CNR) of Rome. Prior to data collection, children's parents received complete information concerning the rationale and effectiveness of EMDR-IGTP, the study procedures, and handed over their written informed consent to allow their child to participate to the research study, as stated in the Declaration of Helsinki.

### EMDR-IGTP procedure

EMDR Therapists administered the EMDR-IGTP to 22 groups, including 7–24 children (Mean = 10.57, Standard Deviation = 7.01), in the schools of Norcia and of other nearby villages. Each group (hereafter referred to as “EMDR-ITGP Group”) had two co-therapists: having two partnered therapists facilitates the management of particularly intense post-traumatic reactions in some children, who might have blocking beliefs, previous traumatic experiences, and/or might require additional time for processing. Each child completed EMDR-ITGP cycles within the same EMDR-ITGP Group.

**Figure 1:**
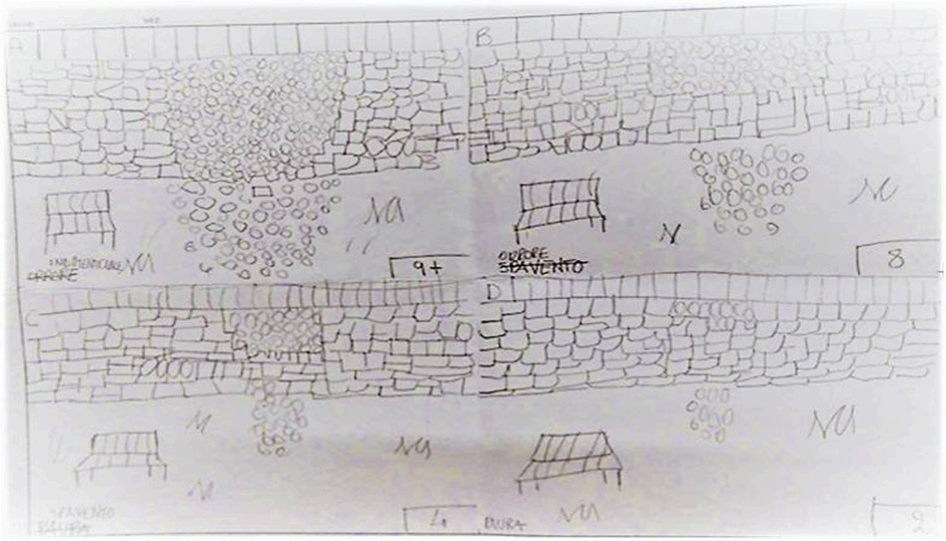
Example of a child's drawings completed during the Assessment and the Desensitization phase of EMDR-IGTP. These drawings have been reproduced with permission from parents.

**Figure 2:**
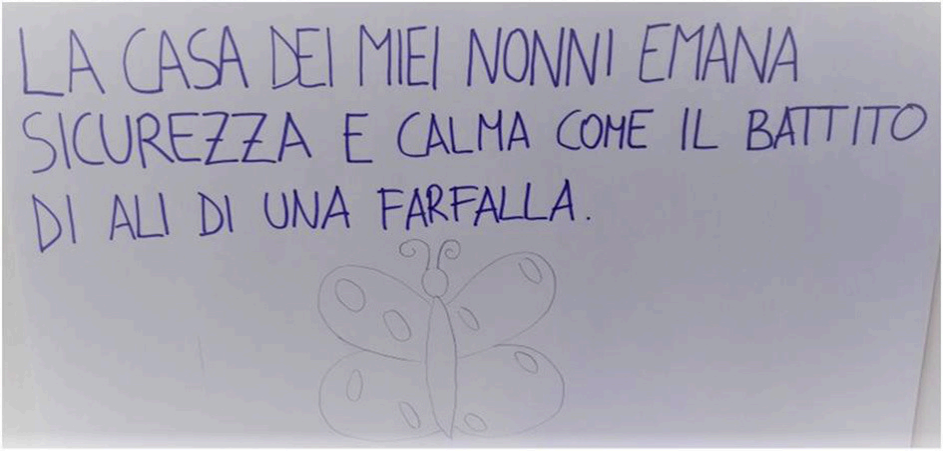
Example of a child's drawing completed during the Installation phase of EMDR-IGTP (translated from Italian to English: “My grandparents' house makes me feel safe and calm, just like the flutter of the wings of a butterfly”). This drawing has been reproduced with permission from parents.

The intervention was conducted according to the recommendations of Shapiro (2001) on EMDR treatment, and following the procedures of a partially modified version of the EMDR-IGTP (Fernandez and Maslovaric, 2016) (Table 1, Figures 1, 2). The therapists used a symptom-focused approach, to identify the most disturbing aspect of the traumatic event, as well as current triggers and related future anxiety. EMDR-IGTP session duration varied from 60 to 90 min, based on the children's development stage, as well as on how they responded.

**Table 1:** Overview of the EMDR-IGTP for children.

**EMDR-IGTP Phases**	**Description**
Phase 1: Client History	It involves history taking, client evaluation, identification of traumatic memories to be targeted, and treatment planning. In this phase, information collected from parents, caregivers and teachers are an essential aspect of the intervention, since they allow to better evaluate the children's “*ability to deal with the high levels of disturbance potentially precipitated by the processing of dysfunctional information. Evaluation therefore involves an assessment of personal stability and current life constrains”* (Shapiro, 2001, p. 70).
Phase 2: Preparation	Children are prepared for treatment, through stabilization procedures and by increasing access to positive affects. This phase is very important for establishing rapport and trust, as well as for facilitating group formation. Children are repeatedly validated regarding their feelings and other post-traumatic symptoms. Subsequently, the team leader instructs children to perform the BH (Artigas et al., 2000) by crossing their arms and alternating tappings on their chest. Children are asked to close their eyes and think of a place where they feel safe or calm, by using their imagination, visualizing the colors and sounds of this “safe place.” At the end of this phase, children are given crayons and paper and are instructed to divide a sheet of the paper in four, marking each square with either A, B, C, and D.
Phase 3: Assessment	Instead of being asked to access the perceptual, cognitive, affective, and somatic components of a specific disturbing memory (as in the standard EMDR protocol), children are asked to think about the most disturbing part of the event (that is, the aspect that made them feel most frightened, angry, or sad), and then draw the image on the paper (see Figure 1, drawing A). Therefore, the critical event (and its associated negative emotions) is not visualized mentally (as in traditional EMDR): it is concretely represented in the children's drawing. Children are asked to rate the level of emotional disturbance elicited of their drawing, referring to a scale from 0 to 10 (where 0 is no disturbance and 10 is worst disturbance), and write the number on the upper right hand corner of the drawing. This provides the team with the children's measures of the Subjective Units of Disturbance (SUD).
Phase 4: Desensitization	Children are asked to focus on the first drawing and on its associated emotions, thoughts and bodily sensations, while simultaneously using the BH (for about 30–60 s, depending on the development stage and the level of affect tolerance). After 3 or 4 BH sets, children are asked to draw a second picture related to the event (in square B), and rate it according to its level of distress. Next, children focus on the second drawing and use the BH. This process is repeated until four drawings are done (Figure 1).
Phase 5: Installation*	Children are asked to focus on the positive memories or bodily sensations they have experienced throughout the BH sets, and then to draw the image on the back of the paper. Children who can't find any positive memory or sensation are asked to draw the place they feel safe in, along with a written word or a written sentence that describes the picture (see Figure 2). The drawing and the word (or sentence) are paired with the BH bilateral stimulation (for about 15–20 s).
Phase 6: Body Scan*	Any residual physical disturbance associated with the memories are processed until children report that the body is clear and free of any disturbance.
Phase 7: Closure*	Children's stability at the end of an EMDR session and between sessions is ensured.
Phase 8: Reevaluation*	At the beginning of the following sessions, therapists assess whether results are maintained or if further reprocessing is needed. In addition to targeting past traumatic experience, EMDR also targets current triggers and related future anxieties.

BH, Butterfly Hug; *phases in which adjustments to the original EMDR-IGTP (Jarero et al., 2012) were introduced by Fernandez and Maslovaric (2016).

### Clinical scales

**Figure 3:**
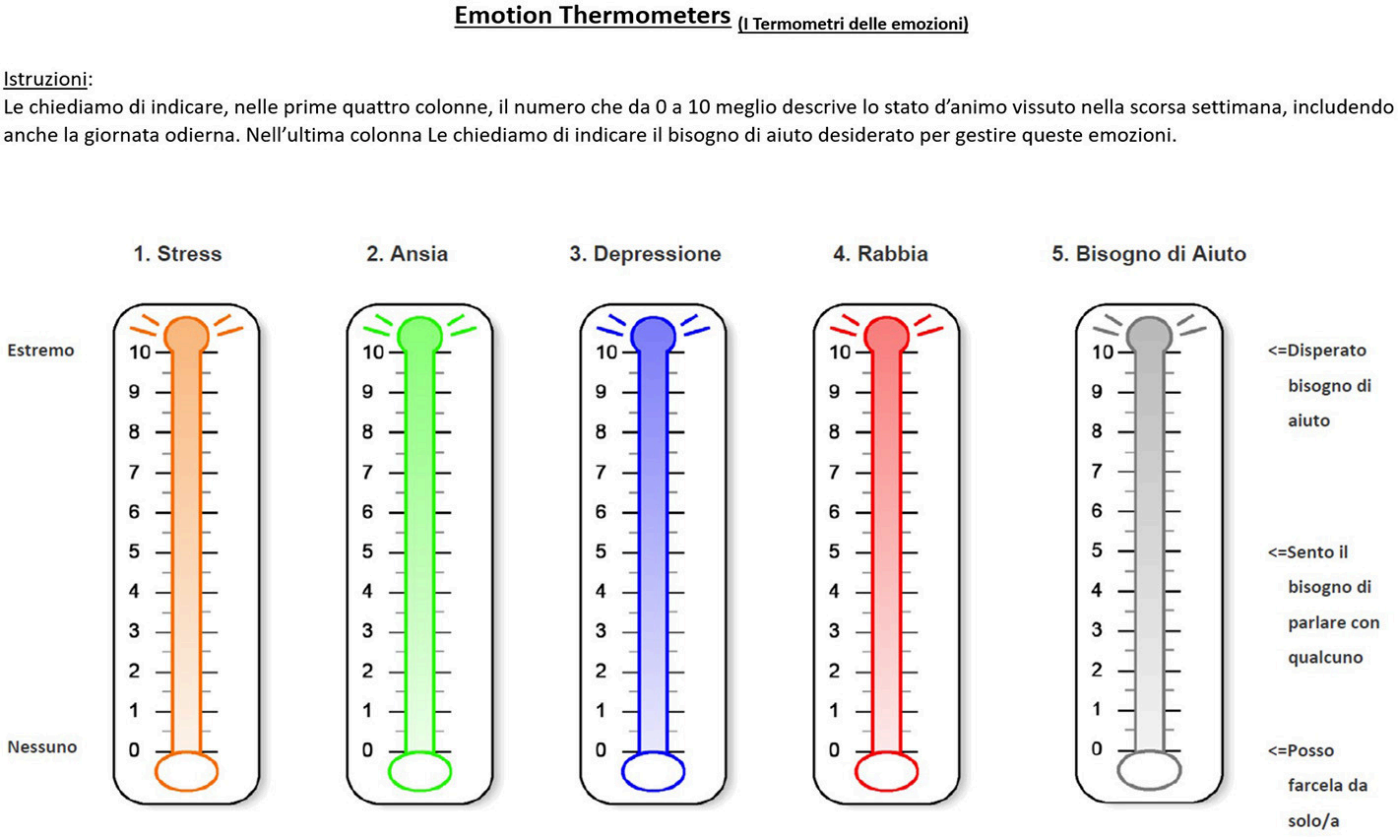
The Emotion Thermometers. Translation from Italian to English: “Istruzioni: Le chiediamo di indicare, nelle prime quattro colonne, il numero che da 1 a 10 meglio descrive lo stato d'animo vissuto nella scorsa settimana, includendo anche la giornata odierna. Nell'ultima colonna Le chiediamo di indicare il bisogno di aiuto desiderato per gestire queste emozioni = Instructions: In the first four columns, please mark the number (0–10) that best describes how much emotional upset you have been experiencing in the past week, including today. In the last column please indicate how much you need help for these concerns.” “Distress,” “Distress;” “Ansia,” “Anxiety;” “Depressione,” “Depression;” “Rabbia,” “Anger;” “Bisogno di aiuto,” “Need Help;” “Disperato bisogno di aiuto,” “Desperately;” “Sento il bisogno di parlare con qualcuno,” “Need to talk with someone;” “Posso farcela da solo/a,” “Can manage by myself”.

The Emotion Thermometers [ET-5; Mitchell et al., 2010; Italian translation, retrieved from http://www.psycho-oncology.info/ET.htm] is a widely used tool for the detection and monitoring of emotional disorders. ET-5 includes single-item five scales, providing rapid and reliable measures of four emotional domains (distress, anxiety, depression, anger) and one non-emotion domain (need for help). Each scale is a graphic thermometer chart, which includes the Distress Thermometer, the Anxiety Thermometer, the Depression Thermometer, the Anger Thermometer, and the Need Help Thermometer (Figure 3). Each domain is rated on an 11-point Likert scale, ranging from 0 (None) to 10 (Extreme), based on the level of emotional distress experienced during the past week. This is an easy to use tool in a post disaster and field study context, with a simple scoring system. The Children's Revised Impact of Event Scale (CRIES-13; Perrin et al., 2005) is a 13-item scale adapted from the Impact of Event Scale (IES; Horowitz et al., 1979), widely used to screen children at high risk for PTSD. Items are rated on a 4-point Likert scale (None = 0, Rarely = 1, Sometimes = 3, and A lot = 5), according to the frequency of recurrence of post-traumatic stress reactions during the past week, as well as in relation to a specific traumatic event noted at the top of the scale. The total score ranges from 0 to 65 and is obtained from the scores on three subscales: Intrusion (four items), Avoidance (four items) and Arousal Symptoms (five items). In this study, only Total score was used in the analyses. Cronbach's α for CRIES-13 score in this study was α = 0.79.

### Data analysis

Linear Mixed-Model Repeated Measures were conducted to assess reduction in the severity of emotional problems and post-traumatic symptoms in children over time, assuming pre- and post-EMDR-IGTP as a *Within-Subject* factor (T0 and T3, respectively). Time elapsed from the second earthquake and the administration of EMDR-IGTP (hereafter referred to as “Time elapsed”), Age and Gender were entered as covariates in the analyses to check for their modulation of a *Within-Subject fixed effect* of treatment as well as for identifying systematic *Between-Subjects fixed effects*. EMDR-ITGP Group was a covariate to control for the possible *random effect* of the clustering of the subjects (that is, the inclusion of children within the respective EMDR-ITGP Groups). *Effect sizes* for Total Model (Cohen's *f* ^2^) and *specific effects* (η^2^_*p*_) were assessed according to Selya et al. (2012) and Olejnik and Algina (2003), respectively.

The analyses were carried out using SPSS 24.0.

## Results

The mean and the standard deviations scores of the dependent variables at T0 and T3 are reported in Table 2.

**Table 2:** Mean (M) and Standard Deviation (*SD*) scores on ET-5 and CRIES-13 at pre and post EMDR-IGTP in children.

**Clinical measures**	**T0**		**T3**	
	***n***	**M (*SD*)**	***n***	**M (*SD*)**
Distress Thermometer	332	3.84 (3.72)	265	3.01 (3.41)
Anxiety Thermometer	332	5.11 (3.92)	264	1.39 (1.95)
Depression Thermometer	332	3.62 (3.65)	266	2.70 (3.33)
Anger Thermometer	332	4.33 (4.02)	263	4.33 (4.10)
Need Help Thermometer	332	3.92 (3.72)	265	3.28 (3.71)
CRIES-13	332	20.21 (17.63)	323	9.88 (13.71)

**Figure 4:**
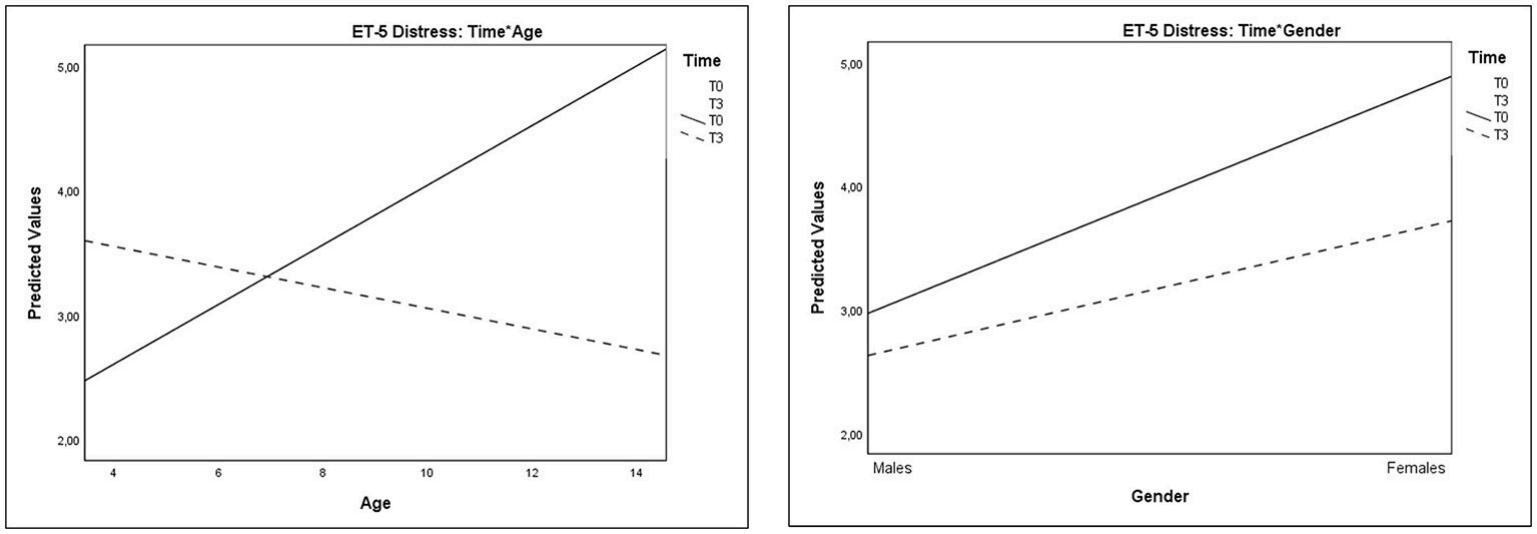
Plots showing significant interactions of Time*Age and Time*Gender for Distress Thermometer scores.

As regards the Distress Thermometer, analyses revealed a marginally significant main effect of Time and significant interactions of Time^*^Age and Time^*^Gender (Table 3 and Figure 4). These results indicated a relevant reduction of distress in older children and a mild increase on this domain in younger ones over time. Moreover, reduction of distress was greater in females than in males.

**Table 3:** Pre vs. post EMDR-IGTP treatment: statistically significant differences on Distress Thermometer scores in children.

**Source of variation**	***F***	***p***	**$\eta_{\text{p}}^{2}$**	**Cohen's *f*^2^**
				0.07
Time	3.845	0.051	0.01	
Time*Time elapsed	0.645	n.s	0.01	
Time*Age	5.604	0.004	0.04	
Time*Gender	10.572	0.001	0.08	

**Figure 5:**
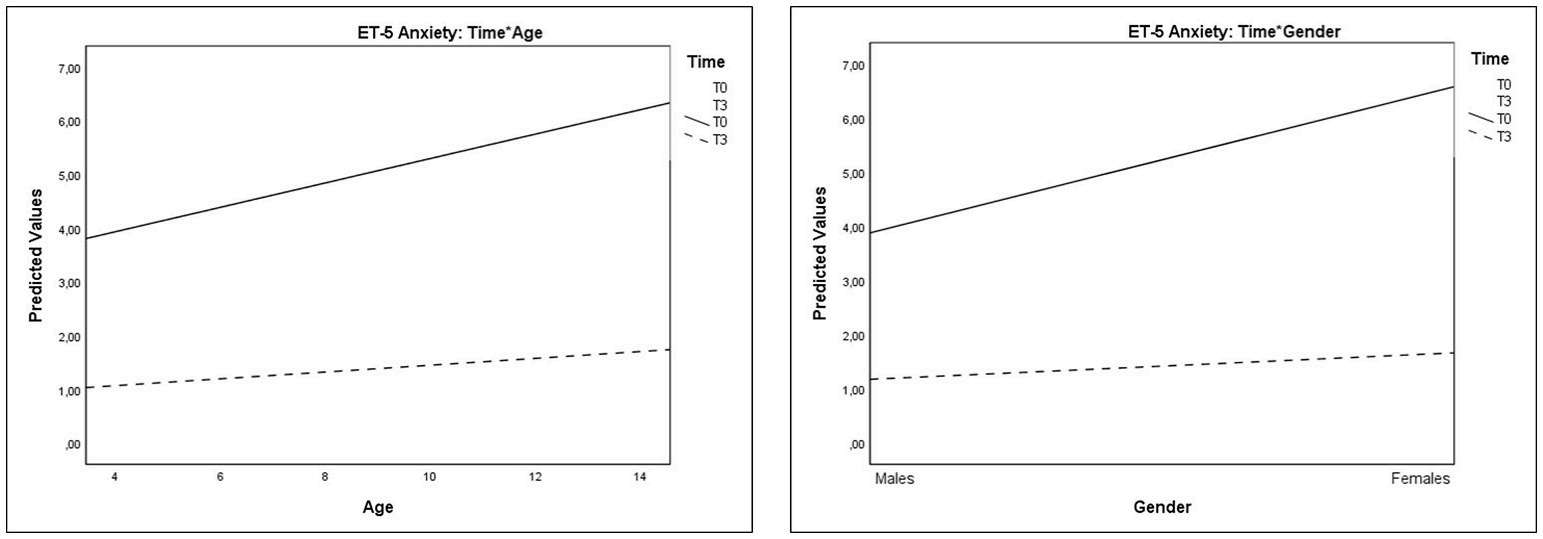
Plots showing significant interactions of Time*Age and Time*Gender for Anxiety Thermometer scores.

Significant interactions of Time^*^Age and Time^*^Gender were also observed on Anxiety Thermometer scores (Table 4 and Figure 5). These results evidenced that the decrease of anxiety from T0 to T3 was greater in older children than in younger ones and greater in females than in males.

**Table 4:** Pre vs. post EMDR-IGTP treatment: statistically significant differences in Anxiety Thermometer scores in children.

**Source of variation**	***F***	***p***	**$\eta_{\text{p}}^{2}$**	**Cohen's *f*^2^**
				0.47
Time	1.100	n.s.	0.00	
Time*Time elapsed	0.936	n.s	0.01	
Time*Age	3.544	0.030	0.03	
Time*Gender	17.708	0.001	0.12	

**Figure 6:**
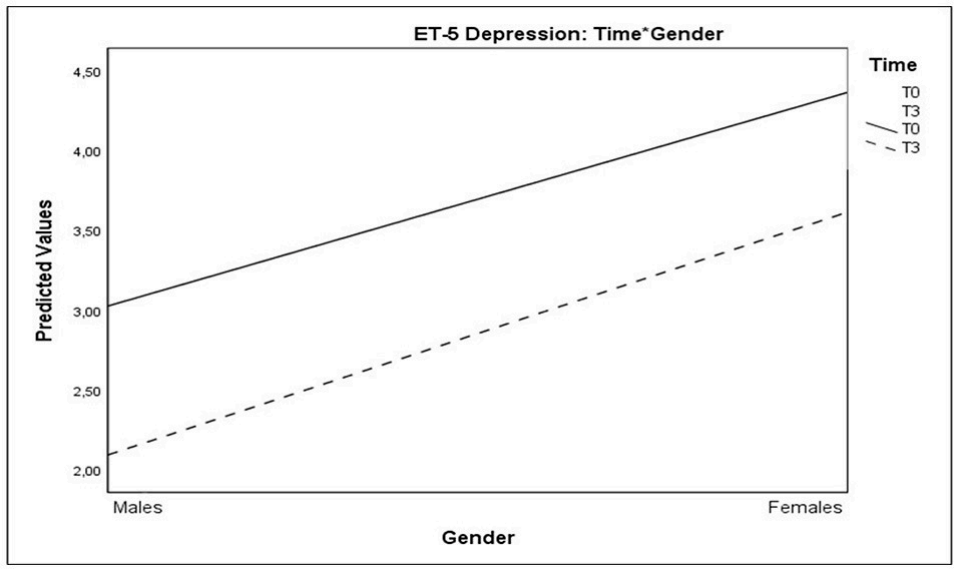
Plots showing significant interactions of Time*Gender for Depression Thermometer scores.

As regards the Depression Thermometer scores, analyses showed significant Time^*^Gender interaction, indicating an improvement in depressive symptoms, which was more evident in males than in females (Table 5 and Figure 6).

**Table 5:** Pre vs. post EMDR-IGTP treatment: statistically significant differences in Depression Thermometer scores in children.

**Source of variation**	***F***	***p***	**$\eta_{\text{p}}^{2}$**	**Cohen's *f*^2^**
				0.05
Time	0.697	n.s	0.00	
Time*Time elapsed	0.729	n.s	0.01	
Time*Age	2.338	n.s	0.02	
Time*Gender	7.218	0.001	0.05	

**Figure 7:**
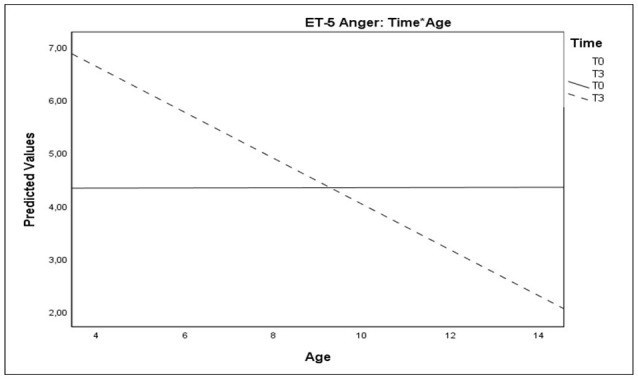
Plots showing significant interactions of Time*Age for Anger Thermometer scores.

As regards the Anger Thermometer scores, analyses evidenced a significant main effect of Time and a significant interaction effect of Time^*^Age (Table 6 and Figure 7). These results evidenced that, at T3, older children showed a reduction of anger, whereas younger children showed an increase on this domain.

**Table 6:** Pre vs. post EMDR-IGTP treatment: statistically significant differences in Anger Thermometer scores in children.

**Source of variation**	***F***	***P***	**$\eta_{\text{p}}^{2}$**	**Cohen's *f*^2^**
				0.04
Time	9.738	0.002	0.04	
Time*Time elapsed	1.188	n.s.	0.01	
Time*Age	9.581	0.001	0.07	
Time*Gender	0.374	n.s.	0.00	

**Figure 8:**
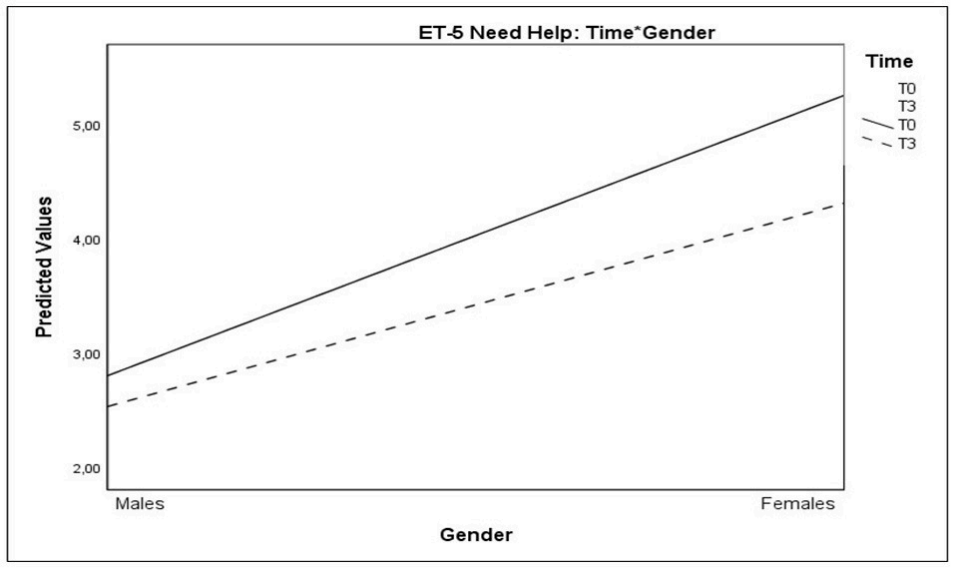
Plots showing significant interactions of Time*Gender for Need Help Thermometer scores.

A significant interaction effect of Time^*^Gender was observed on Need Help Thermometer: these results indicated that the decrease of need for help was more relevant in females than in males (Table 7 and Figure 8).

**Table 7:** Pre vs. post EMDR-IGTP treatment: statistically significant differences in Need Help Thermometer scores in children.

**Source of variation**	***F***	***p***	**$\eta_{\text{p}}^{2}$**	**Cohen's *f*^2^**
				0.08
Time	1.373	n.s.	0.01	
Time*Time elapsed	0.194	n.s.	0.00	
Time*Age	2.507	n.s.	0.02	
Time*Gender	15.479	0.001	0.11	

**Figure 9:**
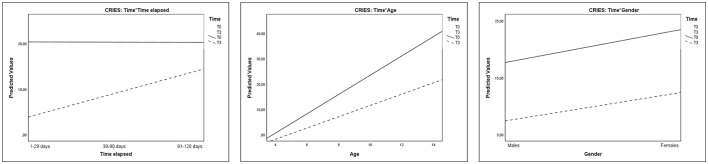
Plots showing significant interactions of Time*Time elapsed, Time*Age, and Time*Gender for CRIES scores.

As regards the CRIES scores, analyses evidenced a reduction of post-traumatic reactions in children from T0 to T3, that resulted to be significantly associated with the time that had elapsed since the second earthquake and since the administration of EMDR-IGTP treatment (Time^*^Time elapsed), with children's age, and with children's gender (Table 8 and Figure 9). These results evidenced that the reduction of post-traumatic symptoms increased in children who had received treatment earlier. Moreover, such improvement was greater in older children than in younger ones and marginally greater in females than in males.

**Table 8:** Pre vs. post EMDR-IGTP treatment: statistically significant differences in CRIES-13 scores in children.

**Source of variation**	***F***	***P***	**$\eta_{\text{p}}^{2}$**	**Cohen's *f*^2^**
				1.19
Time	2.287	n.s.	0.01	
Time*Time elapsed	17.331	0.001	0.12	
Time*Age	72.186	0.001	0.36	
Time*Gender	5.693	0.004	0.04	

Analyses revealed no significant *random effect* of EMDR-ITGP Group, for both ET-5 Thermometers and CRIES scores. Model *effect size* (Cohen's *f*^2^) approached the very large threshold for CRIES, the large threshold for Anxiety Thermometer, the small-medium threshold for Distress and Need Help Thermometers, and the small threshold for Depression and Anger (Cohen, 1988) (Tables 3–8).

## Discussion

Research has largely documented the dramatic effects of natural disasters among children and adolescent survivors. Even though the reported prevalence rates of symptoms significantly vary across studies (Wang et al., 2013), PTSD, anxiety, and depression are commonly observed in the exposed population (Liu et al., 2011; Zhang et al., 2011), in conjunction with other forms of emotional distress (Toyabe et al., 2006; Oyama et al., 2012) and severe difficulties in regulating anger (Durkin, 1993; Kar and Bastia, 2006; Becker-Blease et al., 2010; Forbes et al., 2015).

Coherently with such premises, in this preliminary study the ET-5 and the CRIES-13 was used to investigate the effects of EMDR-ITGP on emotional disorders and post-traumatic symptoms in children who experienced both earthquakes that hit central Italy in 2016. The ET-5 and the CRIES-13 have been implemented in this study to obtain valid measures of psychopathological symptoms in children, with the aim of providing them with immediate help and support, and to restore rapidly their psychological adaptive functioning. The ET-5 is a simple rapid modular screening tool that is widely used for the detection and the monitoring of emotional disorders, both in clinical and research practice. The simple visual-analog thermometer format on which the 5 scales (distress, anxiety, depression, anger, and need for help) are presented is particularly easy to understand for children, quick to administer and simple to score (Mitchell et al., 2010). The CRIES-13 is an easy to understand self-report instrument, which has been specifically designed to identify children with PTSD using the minimum number of items necessary to accurately detect this disorder. The CRIES-13 utility in the screening of post-traumatic distress has been largely documented in tens of thousands of children around the world, in the aftermath of natural disasters (Perrin et al., 2005).

After the conclusion of the intervention, older children showed a reduction of distress and anger, whereas younger children reported an increase on these domains; moreover, older children reported a greater reduction of anxiety than younger ones. A greater reduction of distress, anxiety, and need for help was evidenced in females, whereas a greater improvement in depressive symptoms was evidenced in males. The effects of the EMDR-IGTP treatment on post-traumatic symptoms were particularly evident in older children, compared to younger ones, and marginally greater in females than in males; moreover, a greater improvement was found in children who had received a timelier intervention, than in those who received delayed treatment.

The results of this study provide further evidence for the contribution of EMDR procedures in restoring psychological functioning in child survivors of natural disasters (Grainger et al., 1997; Chemtob et al., 2002; de Roos et al., 2011; Tang et al., 2015).

It may be assumed that children coping strategies are more vulnerable to the overwhelming effects of a disaster compared to adults (Norris et al., 2002b), which makes children particularly sensitive to early psychological support. On the other hand, research in clinical settings has documented, that stress reactions in children are not only very different from those manifested by adults, but also vary according to their age (Şalcioğlu and Başoğlu, 2008). In preschoolers, the severity and manifestation of post-traumatic stress is strictly linked to the emotional reactions of their primary caregiving system, to their parent's ability to face trauma, as well as to the latter's ability transmit to their child a sense of safety and security (Green et al., 1991). Young children show less emotional numbing (Eth and Pynoos, 1985), and tend to manifest persistent reactivity toward a variety of stimuli which may not be directly associated with the original trauma, as they lack the capacities to recognize and regulate strong emotions (Schwarz and Perry, 1994). As a result, young children who experience trauma are consistently unable to adequately monitor their behaviors (Dodge, 1995), experience more anger, and display more overt aggression with parents and peers (Perrin et al., 2000; Vigil-Colet and Codorniu-Raga, 2004; Cohen et al., 2010). Traumatic events are commonly re-experienced through repetitive and compulsive play, in which trauma is reenacted, and/or through drawings that realistically depict some specific aspects of the traumatic experience(s) (Scheeringa et al., 1995). Moreover, young children's ability to put into words avoidance reactions is significantly hampered by their limited capacity for complex cognitive introspection. As a result, it is very difficult to accurately diagnose PTSD in young children according to the current Diagnostic and Statistical Manual of Mental Disorders (DSM-5; American Psychiatric Association, 2013). On the contrary, older children are more likely to manifest post-traumatic responses similar to those seen in adults (Cohen et al., 2010), because of their higher cognitive ability in understanding traumatic events, as well as the consequences from a long-term perspective (Dyregrov and Yule, 2006). In our study, the observed increase of distress and anger in young children at T3 may be ascribed to the contribution of EMDR-ITGP in increasing their ability to correctly identify negative emotions, compared to the pre-treatment phase.

Studies in the field of trauma have documented that, as among adults, gender tends to predict risk for the development of symptomatic distress also during childhood. In fact, female children tend to show higher rates of mood or anxiety symptoms following traumatic stress (Bokszczanin, 2007; Lazaratou et al., 2008), whereas male survivors may show higher rates of behavior symptoms (Shaw et al., 1996). Mechanisms that have been proposed to account for such differences include that female survivors may exhibit more extreme acute reactions to traumatic events and are more likely to use rumination (Hampel and Petermann, 2005): it has been proposed that these reactions may account for an increased risk of developing trauma-related symptoms in females (Udwin et al., 2000; Pine and Cohen, 2002).

The present study provides a clear picture of gender differences in children's response to EMDR-ITGP after a natural disaster. At the end of the treatment, females showed a greater reduction in the severity of emotional problems (that is, distress, anxiety, and need for help) and post-traumatic symptoms compared to males: these findings may be ascribed to the contribution of EMDR-ITGP in restoring psychological functioning in females, by increasing their ability to control acute reactions to traumatic events. On the contrary, females showed a lower improvement in depression compared to males: these results are coherent with those of previous studies, which indicate a greater vulnerability for depressive symptomatology in female children following traumatic stress (Bokszczanin, 2007; Lazaratou et al., 2008). These aspects may be congruent with the *social-cognitive approach*, according to which male and older children tend to report better control of their feelings, compared to females and younger children (Chen et al., 2002; Norris et al., 2002a; Bokszczanin, 2007).

Given this, it might be assumed that, as in the case of post-traumatic stress expression, both age and gender may influence child's treatment responses, especially in the context of a natural disaster, where the environmental disruption can be very challenging to cope with. We believe that these assumptions need to be largely explored by further researches.

The main results of this study highlight the fact that the promptness of treatment may be a key component in restoring a child's post-traumatic reactions.

A very recently published study (Saltini et al., 2017) has explored the effects of EMDR Recent Traumatic Episode Protocol (EMDR R-TEP; Shapiro and Laub, 2008, 2009, 2014; Shapiro, 2012) on post-traumatic distress of acutely traumatized adults, who were exposed to the earthquake that hit Emilia Romagna (a Northern region of Italy) in 2012. The restored psychological adaptive functioning in these subjects was modulated by the treatment provided, not by the time that had elapsed since the traumatic event. These results are coherent with the findings reported by Konuk et al. (2006), who evaluated the effects of EMDR on PTSD symptoms in adult survivors of the 1999 Marmara, Turkey, earthquake.

The above-mentioned findings are very different from those of the present investigation, in which promptness of treatment contributed to post-traumatic symptom reduction in children. There is a large consensus about the importance of early interventions for dealing with the traumatic child responses to natural disaster (Wang et al., 2013; Pfefferbaum et al., 2017). Treatment promptness is fundamental to prevent the worsening of post-traumatic symptoms (Norris et al., 2002b), as well as their persistence across time in children (Honig et al., 1993; Green et al., 1994; Ularntinon et al., 2008; Piyasil et al., 2011).

Psychological interventions are defined as “early” when they are delivered within three months from the traumatic event (Bisson and Cohen, 2006; Bisson and Andrew, 2007; Gibson et al., 2007; Roberts et al., 2009, 2010; Berkowitz et al., 2011). Early psychological interventions are strongly fostered by the American Academy of Child and Adolescent Psychiatry (AACAP) disaster parameter (Pfefferbaum et al., 2013), as well as by the Council of Europe, which established that all European citizens have an equal right to receive psychological support during emergencies.

In a very recent publication, Pfefferbaum et al. (2017) have reported a review of the empirical evidence of early child disaster interventions. This extensive study was done to respond to the “urgent need,” stressed by the National Institute of Mental Health (2002), to establish an evidence base for research on early psychological interventions delivered to exposed children. Only 11 empirical studies (examining 16 early interventions) were identified as eligible for this review, while no empirical investigation of psychological first aid delivered early in the post-disaster phase was found. Among the included studies, only four randomized controlled trials reported improvement on several outcomes, including PTSD, post-traumatic stress symptoms, depression, anxiety, and psychological functioning. Although these results document the effectiveness of the identified interventions, nonetheless, they indicate a lack of evidence of acute interventions in the field of mass disaster contexts.

We believe that these findings should be carefully considered, since they underline the difficulty in promptly planning and carrying out research in the aftermath of a mass disaster, as well as the difficulty in guaranteeing a systematic evaluation of the effectiveness of post-crisis interventions, through canonical research methods. As Wang et al. (2013) posited, “Disaster research is different from most other fields in that much of the work is motivated by a sense of urgency and concern; further, most of the research is theoretical, and little of it is programmatic” (p. 1714). In mass disaster contexts, the priority is to provide help and support, in order to restore rapidly the individual's psychological adaptive functioning. Furthermore, the implementation of systematically designed research is often limited by more urgent needs, such as planning and establishing services for the population.

In this study, the dramatic circumstances of the aftermath, along with the urgent need to provide children with treatment on an equitable basis, precluded the possibility to include a control group. Undoubtedly, this aspect (which is the major constraint of this investigation) raises the question whether the positive changes in children might have been the result of spontaneous recovery, rather than of the EMDR-ITGP treatment. To partially correct this limitation, we also controlled the effect of the elapsed time between the second earthquake and the intervention provision, thus providing a comparison between subjects similar to a wait-list control group. The statistically significant effect of the “Time elapsed” parameter that we found on post-traumatic symptoms may be considered as a *proxy* of a dose-effect relation of EMDR: if an early treatment can be discriminated against a “late” one, it follows that EMDR has an action of its own and that the observed amelioration cannot be ascribed to the pure effect of time.

While scientific literature on the natural course of child PTSD after natural disaster is limited, findings from several researches support the assumption, premised in the present study, that reduced emotional disturbances and post-traumatic symptoms in children, may be the effect of EMDR-ITGP, rather than of spontaneous remission. Several investigations have provided evidence for the PTSD persistence in exposed individuals across time. A long-term follow-up of survivors of the Aberfan disaster in Wales, documented that the levels of PTSD symptoms were still very high 33 years after the critical event (Morgan et al., 2003). In a randomized control trial study, Chemtob et al. (2002) evaluated EMDR effectiveness in child survivors of Hurricane Iniki, Hawaii, who had been previously treated using a school-based, counselor-administered, and brief psychosocial intervention. At a one-year follow-up of the previous intervention, children were still exhibiting significant trauma-related symptoms. In a longitudinal study on the natural course of PTSD in 125 adolescents and young adults, Perkonigg et al. (2005) documented that, 34–50 months after the critical event, nearly half of the subjects reported no significant remission of symptoms. Goenjian et al. (1995) found that 1.5 years after the 1988 Armenian earthquake, 95 percent of children who survived from a severely hit city and 26 percent of others who suffered a less strong earthquake, were still experiencing severe levels of PTSD. Finally, in a study by Carr et al. (1997), the prevalence of PTSD in survivors of the 1989 earthquake in Newcastle, Australia, had only decreased by about 50 percent in the first 2 years after the event (Carr et al., 1997). In line with these empirical evidences, it seems reasonable to state that EMDR-IGTP contributed substantially to the reduction of post-traumatic symptoms in children included in this study. Such results appear particularly relevant, if we consider that EMDR-IGTP was administered to children who had experimented both earthquakes (including ongoing seismic oscillations and aftershocks).

EMDR's effectiveness in the treatment of trauma is now largely documented; its efficacy has received empirical evidence in the field of natural disasters, where the emergency circumstances are a real obstacle for the implementation of experimental designs (Grainger et al., 1997; Chemtob et al., 2002; de Roos et al., 2011; Tang et al., 2015). In this preliminary study, the use of EMDR-ITGP provided a great opportunity to deliver psychological interventions to a great number of exposed children, maximizing the possibility to rapidly deal with the emergency crisis.

## Conclusions

The field of mass disaster psychology has rapidly developed in the past years given the dramatic *sequelae* of natural disasters that have occurred in several places of the world, causing death, disruption, and terror. There is now universal consensus about the importance of early psychological interventions to prevent both the worsening and persistence of trauma-related symptoms in post-disaster survivors, especially in children who are less equipped to cope with the effects of critical events.

Results of this preliminary field study show that EMDR-ITGP contributed significantly in reducing emotional disturbances and post-traumatic symptoms in exposed children, principally (regarding the post-traumatic symptomatology) in those who had received treatment earlier. As we have stated above, the need to respond to the emergency precluded, mainly for ethical reasons, the possibility to include a control group. We believe that this aspect is the major “scientific” limitation of this study (when considering only the compliance factor to the experimental design criteria), yet also its major strength, since the urgent need to treat children on an equitable basis is the main priority of any experimental design.

In the future, more rigorous studies may shed further light on the role of children's age, gender, and other relevant dimensions (e.g., parent's ability to instill a sense of security) in modulating the response to EMDR-ITGP in child survivors of natural disaster.

## Author contributions

CT analyzed data and wrote this paper. As first author, she is primarily accountable for all aspects of the work. ML analyzed data, revised the paper for intellectual content, and approved its final version to be published. AG contributed substantially to data analysis, revised the paper for intellectual content, and approved its final version to be published. GM contributed substantially to the recruitment of subjects, administered EMDR therapy, and acquired psychological data. She revised the paper for intellectual content and approved its final version to be published. RT revised the paper for intellectual content and approved its final version to be published. IF conceived the work, supervised substantially to the recruitment of subjects, provided a substantial contribution to the interpretation of data, and counseled in essential questions about EMDR-ITGP therapy. She revised the paper for intellectual content and approved its final version to be published. MP monitored data acquisition and provided a substantial contribution to the interpretation of data. He revised the paper for intellectual content and approved its final version to be published. ML, AG, GM, RT, IF, and MP agreed to be accountable for all aspects of the work and to ensure that questions related to the accuracy or integrity of any part of the work were appropriately investigated and resolved.

### Conflict of interest statement

The authors declare that the research was conducted in the absence of any commercial or financial relationships that could be construed as a potential conflict of interest.
